# Extended balloon labour induction: A single arm proof of concept trial

**DOI:** 10.1016/j.eurox.2023.100226

**Published:** 2023-08-13

**Authors:** Lorato Matshitsa, Nassali Mercy-Nkuba, G. Justus Hofmeyr

**Affiliations:** a Department of Obstetrics and Gynaecology, University of Botswana, Pvt Bag, 00703 Gaborone, Botswana; b Department of Obstetrics and Gynaecology, University of the Witwatersrand and Walter Sisulu University, East London, South Africa

**Keywords:** Foley catheter balloon, Labour induction, Labour augmentation, Favourable cervix

## Abstract

**Background:**

Mechanical labour induction with a transcervical balloon is as effective as pharmacological methods, with fewer adverse effects. Its use has been limited to labour induction with an unfavourable cervix able to retain the balloon. We have developed an innovative approach using 2–3 balloons side-by-side to extend the benefits of mechanical labour induction/augmentation with a favourable cervix without having to resort to amniotomy or uterotonics.

**Objectives:**

To assess the effectiveness of side-by-side balloons for extended labour induction/augmentation.

**Methods:**

We conducted a single arm proof of concept trial at Princess Marina Hospital (PMH) in Gaborone, Botswana. Participants were assessed as unsuitable for single balloon labour induction based on a modified Bishop cervical score of 7 or more. Three Foley catheters taped symmetrically side by side were passed through the cervix and inflated with 60mls each. Traction was applied with a weight of 250mls water suspended over the bed-end. The use of uterotonics, time from induction to birth, mode of birth, Apgar scores and adverse maternal and neonatal outcomes were recorded. Participants’ satisfaction with the method of labour induction was assessed using a descriptive scale.

**Results:**

We enrolled 20 participants of whom two were nulliparous. Indications for labour induction were mainly late term (70%) and hypertensive disorders of pregnancy (25%). The mean cervical score was 7.2. Overall, 17 (85%) of the participants achieved a vaginal birth, of whom 5 required oxytocin for labour augmentation. Four of these had requested removal of the balloons, one due to discomfort and three felt the process was taking too long. Three participants underwent caesarean birth. The mean time from initiation of labour induction to vaginal birth was 16 h (standard deviation (SD) 8.4) and 20 h for caesarean birth (SD 10.5). There were no 5-minute Apgar scores below 7 nor neonatal admissions. One baby required brief resuscitation. There were no adverse maternal outcomes.

**Conclusion:**

This proof-of-concept study suggests that use of side-by-side balloons in participants with favourable cervix is effective in achieving vaginal birth in most participants without uterotonics or amniotomy. There were no adverse outcomes, though the study was too small to exclude the possibility of rare adverse outcomes. This offers a novel option for labour induction/augmentation, particularly where uterotonics or amniotomy are best avoided such as prior caesarean birth, vertical infection transmission risk and in settings with limited capacity for fetal surveillance. The results clearly justify larger randomized trials to evaluate this novel approach with greater precision. On the basis of the current study we are developing a purpose-designed balloon for extended balloon labour induction.

## 1 Introduction

The rates of labour induction are steadily increasing globally [1]. The WHO Global Survey found that almost one in 10 births involved labour induction. [2] Rates among English hospitals between 2015 and 2017 ranged from 17.5% to 40.7% [3]. This increasing global prevalence justifies investment in research to improve the effectiveness, acceptability and safety of labour induction methods. Mechanical methods such as an extra-amniotic balloon passed through the uterine cervix have been used since the mid 1800’s. [4] The Modern use of the foley catheter balloon for labour induction with an unfavourable cervix was described in 1967 [5], but fell from popularity with the development in subsequent decades of more elegant methods such as dinoprostone and later misoprostol [4]. Several large randomized trials have shown that balloon catheters have similar caesarean birth rates compared with vaginal prostaglandins, and fewer adverse perinatal events [6], [7]. Patient satisfaction has been assessed as high, with a preference for balloon plus misoprostol labour induction over misoprostol alone [8]. Based on evidence of safety, effectiveness and acceptability, wider use of foley catheter induction of labour in the UK has been advocated [9].

The Foley catheter has generally been used for labour induction with an unfavourable cervix. The mechanism of action is thought to include release of endogenous prostaglandins as occurs in spontaneous labour [10]. Effectiveness is improved by increasing the volume with which the balloon is inflated, [11] as well as by weighted traction [12], [13]. The more favourable cervix is unable to retain the Foley balloon, thus requiring prostaglandins, oxytocin and/or amniotomy for labour induction.

Given the increasing evidence of the benefits of balloon labour induction over pharmacological methods, we have reported in this journal the novel concept of ‘extended balloon labour induction’ using two or more Foley balloons side-by-side when the cervix ix too dilated to retain a single balloon [14]. The purpose of this single arm pilot trial was to generate preliminary data on the effectiveness, safety and acceptability of this novel method.

## 2 Theory

Our theory was that ‘extended balloon labour induction’ would result in vaginal birth within 60 h without the need for uterotonics or rupture of membranes in at least 50% of participants.

## 3 Materials and methods

The study was conducted from February 2022 to July 2022 at Princess Marina Referral Hospital (PMH) which is the largest referral hospital in Botswana. PMH also acts as a district hospital for patients living in Gaborone and surrounding areas.

There are over 6000 birth a year in PMH, of which one in 3 are caesarean births.

### 3.1 Study population

Women admitted for induction of labour who met the inclusion criteria and consented to participate in the study.

### 3.2 Sample size determination

As this was the first formal study of this method, it was tested in a limited number of participants (20) to assess feasibility, acceptability and detect any unanticipated adverse effects.

### 3.3 Inclusion criteria

Nulliparous and multiparous women 18 years or older without serious morbidity due for labour induction, who give consent to participate; cervix considered too far dilated to retain a single balloon, or single balloon has fallen out while not yet in labour; modified Bishop cervical score [15] of 7 or more; singleton and cephalic; gestation 37 + 0 weeks or more; estimated fetal weight less than 4 kg.

### 3.4 Exclusion criteria

These were previous caesarean birth or urgent labour induction indications (eg eclampsia, preeclampsia with severe features; ruptured membranes).

### 3.5 Study design

This was a prospective, single arm clinical trial to obtain preliminary evidence of the efficacy of the novel method.

### 3.6 Study procedures

#### 3.6.1 Screening

All records of patients admitted for induction of labour were screened by the first author. Those that met the inclusion criteria were approached**.**

#### 3.6.2 Recruitment

Patients admitted for induction of labour underwent cervical status assessment by the admitting doctor and review of indications. If the admitting doctor found the patient eligible, they contacted the first author who confirmed cervical status. The first author explained the study to those eligible and requested consent to participate in the study using the extended balloon method of induction.

#### 3.6.3 Consent process

The consenting process was conducted in a private room at the antenatal ward. Written consent was signed by both the first author and participant. The participant was given a copy.

#### 3.6.4 Trial procedures

Under sterile conditions, three Foley catheters FG18 with a 30 ml balloon were taped together symmetrically at mid-shaft to maintain the side-by-side orientation of the balloons. The cervix was visualised with a speculum and the balloons were passed through the cervix and inflated with 60 mls of sterile fluid each. Gentle traction was applied with a 250 ml water weight hung over the bed-end. Patient monitoring was done according to hospital procedures by the midwives: maternal and foetal checks at least every 4–6 h if not in established labour and once in labour according to the partograph. Maternal checks included measurement of heart rate, blood pressure, temperature, presence or absence of contractions, per vaginal bleeding and spontaneous rupture of membranes. Foetal checks included heart rate assessment with a doptone or cardiotocography. Any complications were managed by the attending medical team according to hospital protocols. A summarised study protocol was attached to the file of participants to ensure well-coordinated care and for staff to be aware when to inform the first author. After birth, the participants were admitted at the postnatal and were reviewed prior to discharge by the first author to complete documentation of delivery outcomes and the satisfaction assessment. Every participant received a P30 (Euro 2.00) airtime voucher as a token of appreciation for participating in the study.

Data were entered onto a paper clinical record form. Data collected at enrolment included participant demographics, indications for labour induction, date and time of induction. The form was completed postpartum with information that included date and time of birth, mode of birth, Apgar score at 1,5 and 10 min and satisfaction with the procedure. Any adverse maternal outcomes such as maternal infection, postpartum haemorrhage, blood transfusion and neonatal outcomes such as need for resuscitation and admission to neonatal intensive care unit (NICU) were also noted.

#### 3.6.5 Data analysis

Data were double entered onto microsoft excel, checked for accuracy and analysed using Epi Info statistical package. Continuous data was reported as mean, median, standard deviation (SD) and categorical data as proportions with percentages.

#### 3.6.6 Ethical aspects

Ethical clearance was obtained from the University of Botswana Institutional Review Board (reference UBR/RES/IRB/BIO/GRAD/145), Ministry of Health and Wellness Research Unit (reference HPDME 13/18/1 18 Aug 2021) and Princess Marina Hospital Research Unit and Ethical Committee (reference PMH 2/2 A(7)/120). Informed consent was obtained from the study participants (written in Setswana or English depending on participants’ preference). Information included that participation was voluntary and could be withheld or withdrawn at any time without giving reasons and this would not prejudice the care the participant received. The trial was registered prospectively on-line on the Pan-African Clinical Trials Registry (PACTR) which is the WHO-approved trails registry for Sub-Saharan Africa. The registration was not processed by PACTR because they did not accommodate single arm trials. The trial was subsequently retrospectively registered on Clinicaltrials.gov (NCT05885087).

## 4 Results

We included 20 participants in the study. The demographic characteristics of the participants are shown in Table 1. Most participants were multiparous. Indications of labour induction were late term pregnancy, hypertensive disorders or both. The pre-induction modified Bishop cervical scores ranged from 7 to 8.

**Table 1:** Maternal Characteristics.

	N	Mean	Standard Deviation	Range
Age (years)	20	32.6	4.9	24–42
Gestation (days)	20	286	6.8	269–294
Nulliparous	2/20 (10%)			
Multiparous	17/20 (85%)			
Grand-multiparous	1/20 (5%)			
HIV Negative	17/20 (85%)			
HIV positive	3/20 (15%)			
Weight(kg)*	20	82	14.8	55–109
Modified Bishop Score*	20	7.2	0.4	7–8
Indication for labour Induction				
-Hypertension	5/20 (25%)			
-Late term	14/20 (70%)			
-Hypertension and late-term	1/20 (5%)			

The intended traction during the induction period was not consistently applied. Some participants opted to place the 250 ml fluid weight on their bed instead of letting it hang over the end of the bed.

**Fig. 1:**
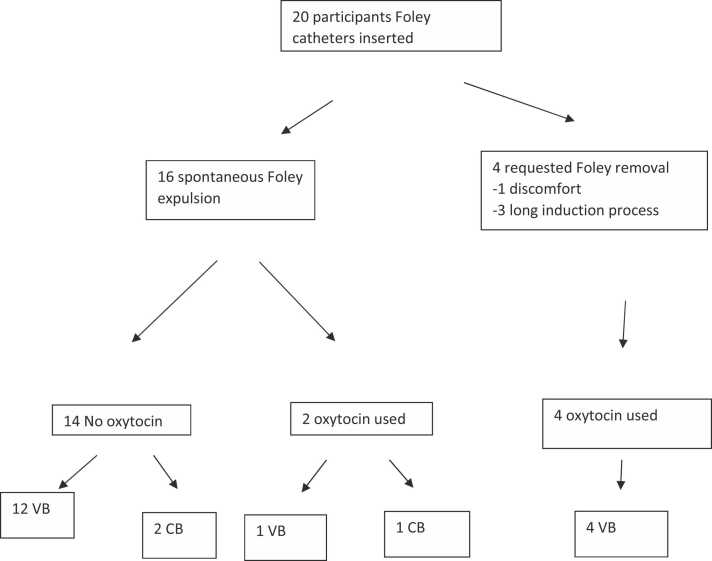
**Study flow Chart,** VB = vaginal birth; CB = caesarean birth.

The participant flow is shown in Fig. 1 and outcomes in Table 2. Overall, 17 (85%) of the participants achieved a vaginal birth following extended balloon induction with 5 of the 17 requiring oxytocin for labour augmentation. Of these 5, 4 had requested balloon removal before spontaneous expulsion due to discomfort (1, after 6 h) or participant concern about the duration of labour (3, after 15, 18 and 19 h).

**Table 2:** Outcomes expressed as mean values, standard deviation and range or proportions (%).

		Vaginal birth (n = 17)				Caesarean birth (n = 3)		
	N	Mean / n	SD/%	Range	N	Mean / n	SD/%	Range
Cervical dilatation (cm) upon removal of Foley on request	4	3.8	0.5	3–4	0			
Cervical Dilatation (cm) upon spontaneous Foley expulsion	13	5.8	1.6	4–10	3	4	1	3–5
Oxytocin augmentation following spontaneous Foley expulsion	13	1/13	7.7%		1	1/3	33%	
Cervical dilatation before oxytocin or caesarean	5	4.2	1.1	3–6	3	4.7	1.5	3–6
Time from induction to birth (hours)	17	15.8	8.4	3.5–28.5	3	20.1	10.5	9.3–30.3
Need for neonatal Resuscitation	17	1/17	5.9%		3	0/3	0%	

SD = standard deviation; 5-minute Apgar score < &: 0; Neonatal intensive care admission: 0

Three participants underwent caesarean birth and the indications were fetal distress and poor progress. The mean time from initiation of induction of labour to delivery was 16 h for vaginal birth and 20 h for caesarean birth.

### 4.1 Neonatal adverse outcomes

None of the participants experienced uterine hyperstimulation during labour. There were no 5-minute Apgar scores below 7. One baby required brief neonatal resuscitation and none was admitted to neonatal intensive care unit.

### 4.2 Maternal adverse outcomes

One participant who gave birth vaginally had post-partum haemorrhage with estimated blood loss of 500 mls and did not require blood transfusion. No puerperal infections were recorded. One participant had intrapartum respiratory infection and tested positive for COVID. This infection was considered unrelated to the study intervention.

### 4.3 Maternal satisfaction

The results of the satisfaction questionnaires are shown in Table 3. Maternal experience was reported as acceptable discomfort at insertion (9), painful but acceptable (10) or unbearable (1). Overall satisfaction with the induction process was reported as acceptable (9) or bearable (11).

**Table 3:** Participants satisfaction (N = 20).

	N (%)
Discomfort during insertion:	
Acceptable	9 (45%)
Painful but acceptable	10 (50%)
Unbearable	1 (5%)
Overall satisfaction with Foley induction process	
Acceptable	9 (45%)
Bearable	11 (55%)
Unbearable	0 (0%)

## 5 Discussion

### 5.1 Effectiveness

The vaginal birth rate (85%) is comparable to that found in the WHO Global Survey of 83.4% in Africa and 81.6% in Asia [16]. The avoidance of uterotonics or amniotomy in 60% is a clinically meaningful benefit, given that all would have required uterotonics and/or amniotomy without the option of ‘extended foley balloon’ labour induction. The request of 4 participants for removal of the balloons may have been influenced by anxiety or uncertainty due the fact that the process was communicated as an experimental approach. In the setting of routine use of this method, birth without use of uterotonics or amniotomy might be more frequent.

### 5.2 Neonatal outcomes

The finding of no 5-minute Apgar scores below 7 or neonatal intensive care unit admissions was reassuring, though safety cannot be assured in a trial of this size. Randomized trials have consistently found more favourable perinatal outcomes for balloon than pharmacological labour induction methods.

### 5.3 Maternal outcomes

Maternal outcomes were also reassuring with only one participant developing PPH which was controlled on uterotonics and did not require blood transfusion and no endometritis was noted prior to discharge. A Cochrane review of 33 trials using the Foley catheter concluded that there is no evidence that balloon catheters increase infection risk [17].

### 5.4 Satisfaction

Most of the participants found the procedures acceptable, but there was one participant who found the discomfort during insertion of the balloons unbearable. The satisfaction responses may also have been influenced by anxiety associated with participation in a trial of an experimental procedure. A randomized trial comparing a double balloon plus misoprostol with misoprostol alone found a preference for the method including the balloon, which also had a positive impact on the overall birth experience [8].

### 5.5 Strengths

This was a prospective trial following a prescribed protocol and all participants were assessed with consistent pre-induction cervical scoring and insertion of Foley catheters by the first author.

### 5.6 Limitations

Interpretation of results needs to be guarded as this was a preliminary pilot study with limited sample size and no comparison group.

## 6 Conclusions

This study suggests that use of ‘extended balloon labour induction’ in participants with favourable cervix is effective in achieving vaginal birth within twenty-four hours in the majority of participants without use of oxytocin or amniotomy. No safety concerns were apparent, though larger trials are needed to confirm safety. Evidence from single balloon randomized trials have shown that balloon labour induction is safer than pharmaceutical labour induction. This offers an option for labour induction without uterotonics or amniotomy when the cervix is too open to retain a single balloon, with the potential to reduce pharmacologically associated uterine hyperstimulation. These benefit s would be relevant to all clinical settings, and more so in pateints with risk factors such as prior caesarean birth or settings with limited capacity for fetal surveillance.

The findings clearly justify further research including randomized trials to determine with greater certainly and precision the potential benefits of extended balloon labour induction.

## Declaration of Generative AI and AI assisted technologies in the writing process

None.

## Funding

None.

## CRediT authorship contribution statement

LPM was responsible for recruiting and consenting of the participants, insertion of foley catheters, data collection and analysis, and wrote the first draft of the protocol and the paper. GJH conceived the labour induction method and the study. NM-N and GJH contributed to supervision and writing and all authors approved the final version of the manuscript.

## Declaration of Competing Interest

None.
